# Validity of the EQ-5D-5L questionnaire among the general population of Poland

**DOI:** 10.1007/s11136-020-02667-3

**Published:** 2020-10-24

**Authors:** Katarzyna Młyńczak, Dominik Golicki

**Affiliations:** 1 Department of Experimental and Clinical Pharmacology, Medical University of Warsaw, 1b Banacha St, 02-097 Warsaw, Poland; 2 HealthQuest spółka z ograniczoną odpowiedzialnością Sp. K, Warsaw, Poland

**Keywords:** EQ-5D, EQ-5D-5L, EQ-5D-3L, Validity, Health-related quality of life, General population

## Abstract

**Purpose:**

We aim to compare the psychometric properties of the EQ-5D-5L questionnaire with the EQ-5D-3L version and EQ VAS, based on a survey conducted in a sample representing the general adult population of Poland.

**Methods:**

The survey comprised health-related quality of life (HRQoL) questionnaires: EQ-5D-5L, EQ VAS, SF-12 and EQ-5D-3L, together with demographic and socio-economic characteristics items. The EQ-5D index values were estimated based on a directly measured value set for Poland. The following psychometric properties were analysed: feasibility, distribution of responses, redistribution from EQ-5D-3L to EQ-5D-5L, inconsistencies, ceiling effects, informativity power and construct validity. We proposed a novel approach to the construct validity assessment, based on the use of a machine learning technique known as the *random forest* algorithm.

**Results:**

From March to June 2014, 3978 subjects (aged 18–87, 53.2% female) were surveyed. The EQ-5D-5L questionnaire had a lower ceiling effect compared to EQ-5D-3L (38.0% vs 46.6%). Redistribution from EQ-5D-3L to EQ-5D-5L was similar for each dimension, and the mean inconsistency did not exceed 5%. The results of known-groups validation confirmed the hypothesis concerning the relationship between the EQ-5D index values and age, sex and occurrence of diabetes.

**Conclusions:**

The EQ-5D-5L, in comparison with its EQ-5D-3L equivalent, showed similar or better psychometric properties within the general population of a country. We assessed the construct validity of the questionnaire with a novel approach that was based on a machine learning technique known as the *random forest* algorithm.

## Introduction

Assessment of the health-related quality of life (HRQoL) is an essential step in the process of inferring the cost-effectiveness of medical technology. To ensure the correctness of pharmacoeconomic inference, the HRQoL estimates should tend to the true value. However, this is difficult because the HRQoL is a latent variable and cannot be measured and interpreted directly. The assessment of psychometric properties, i.e. validation, includes determining if there are grounds to believe that the questionnaire used to assess HRQoL measures what is intended [1]. Therefore, before the questionnaire is included in a study, it should be validated. It means checking to what extent, in a given clinical situation and a specific population, the results of the questionnaire can be correctly interpreted.

The most commonly assessed and pointed in the COSMIN checklist (COnsensus-based Standards for the selection of health status Measurement INstruments) psychometric properties comprise validity, reliability and sensitivity [2]. Validity determines whether the questionnaire measures what is intended to measure and that it is useful for its intended purpose. Reliability assesses whether the results of the questionnaire specifying the same feature will be repeatable for subsequent measurements. Sensitivity is the ability to measure differences in the HRQoL among patients or patient groups [1, 3].

The EQ-5D is one of the most commonly used HRQoL questionnaires in clinical and economic evaluations of health care [4, 5]. It was developed by the EuroQol group in 1990 [6]. The EQ-5D is a generic instrument: a non-specific questionnaire, which has no restrictions on the characteristics of the population for its use. This makes it possible to compare the HRQoL results of assessment within different populations. Each EQ-5D instrument comprises a descriptive system and a visual analogue scale (EQ VAS). The EQ VAS is a typical, thermometer-like, 20-cm-long scale, grading from 0 (representing “The worst health you can imagine”) to 100 (representing “The best health you can imagine”). The descriptive system consists of five dimensions: mobility, self-care, usual activities, pain/discomfort and anxiety/depression. The patient uses it to assess their health with a three-level or five-level scale (EQ-5D-3L and EQ-5D-5L, respectively) [6, 7]. The best health state is determined by a pattern of 11111. A single summary score (EQ-5D index value) may be calculated for the results of the descriptive system (health states profiles). It represents the utility related to the described health state. The utilities are determined based on social preference weights. The EQ-5D index values of health states are then used in economic analyses as part of health technology assessment [8].

In the beginning, the EQ-5D-3L provided the assessment of 243 (i.e. 3^5^) possible health states. Many studies have shown the validity and reliability of the EQ-5D-3L, both in disease-specific (cardiovascular disease [9], schizophrenia [10], rheumatic disease [11], paediatric population [12], cervical cancer [13], diabetes [14]) and general populations [15, 16]. When the use of the EQ-5D questionnaire became more widespread, it was noted that it is necessary to improve its discriminatory power and reduce the ceiling effect (i.e. an unexpectedly increased frequency of the best (“no problem”) health status). This issue was of particular importance in the general population studies [17–22]. Hence, the EuroQol group decided to develop a new version of the questionnaire (EQ-5D-5L), which could cover 3125 (i.e. 5^5^) possible health states [7, 23, 24].

Numerous literature references indicate better, or at least comparable, properties of the EQ-5D-5L version of the questionnaire in comparison to the EQ-5D-3L one [25–28]. As indicated by Buchholz et al. [27], most of the study populations are disease specific: inpatient rehabilitation [29], orthopaedics [30, 31], stroke [32, 33], liver disease [34–36], cancer [37], lung cancer [38], diabetes [39, 40], dermatology [41], psoriasis [42] and mixed population [43]. Recently published studies comparing EQ-5D-3L and EQ-5D-5L also refer to the disease-specific subpopulations: cataract [44], Crohns disease [45] and hip replacement surgery [46]. However, disease-specific populations characterise by predictively worse health and resulting HRQoL, so the distribution of assessed health states in these groups is shifted compared to the distribution in the general population. It shows that it is difficult to transfer the results of a validation study in the disease-specific population to the general one.

In this study, we aim to assess the psychometric properties of the EQ-5D-5L questionnaire in relation to the results of the EQ-5D-3L, and along with the EQ VAS results, based on a survey conducted in a sample representing the general adult population of Poland.

## Methods

### Population and study setting

To obtain a representative sample of the adult population, the administrative area of Poland was divided into 65 strata (16 voivodeships [counties] with 3–9 smaller areas based on the number of inhabitants in each). The previously defined size of the target population of the study was split proportionally into strata. The first stage of random stratified sampling was to take the samples from cities and villages, and then from randomly selected smaller areas (one or several adjacent streets) in previously selected locations. Finally, based on personal identification numbers (PESEL), samples of 8 people with different addresses from each stratum were selected. The maximum error in estimating the frequency of a given category in the sample was 1.55%.

The surveys were conducted with the support of the market research company—Centre for Public Opinion Research (CBOS)—from March to June 2014. The interviewers had to make at least three attempts to contact the respondent. No substitutes were permitted. As part of the survey, respondents answered questions about demographic characteristics and socio-economic status (computer-assisted personal interview, CAPI) and completed by themselves paper-and-pencil polish versions of HRQoL questionnaires in the following fixed order: EQ-5D-5L, EQ VAS, SF-12 and EQ-5D-3L. We also introduced in the survey the additional question about diabetes (the results of the planned substudy will be described elsewhere). A total of 10% of interviews underwent quality control. The Bioethics Committee at the Medical University of Warsaw approval was obtained for the survey (AkBE/34/17).

### Data analysis

The validation of the EQ-5D-5L questionnaire consisted of an analysis of the following psychometric properties: feasibility, distribution of responses, redistribution from EQ-5D-3L to EQ-5D-5L, inconsistencies, ceiling effect, informativity power and construct validity.

We assessed feasibility by calculating the proportion of missing values for the results of the EQ-5D-3L and EQ-5D-5L questionnaires. We tabulated the responses from the descriptive system of EQ-5D-3L and EQ-5D-5L and compared the redistribution. We defined the inconsistency according to Janssen [25] as the proportion of response pairs (from EQ-5D-3L to EQ-5D-5L, all 15 pairs) which does not comply with the scheme: 3L_1_ to 5L_1_ or 5L_2_, 3L_2_ to 5L_2_, 5L_3_ or 5L_4_ and 3L_3_ to 5L_4_ or 5L_5_ (level 1 from EQ-5D-3L to level 1 from EQ-5D-5L, and so on)—the remaining subgroups are considered inconsistent. The consistent responses pairs are in Table 2. We estimated the EQ-5D index values for both versions of questionnaires based on directly measured value sets for Poland [47, 48].

The ceiling effect is defined as the proportion of respondents with either a “no problem” answer for a dimension or with the best health state (11111) [49]. We hypothesise that the ceiling effect for the EQ-5D-3L version will be higher than for EQ-5D-5L. In the general population, in which the expected percentage of patients with the best health will be higher than in the disease-specific population, it is essential to use a questionnaire as resistant to the ceiling effect as possible.

Informativity power determines the degree of uniform distribution of responses in each dimension. The more evenly the answers are distributed, the more useful the questionnaire is. As a measure of informativity power, we calculated the Shannon index (‘*H*’) and the Shannon Evenness index (‘*J*’) [50]. The Shannon index (‘*H*’) is defined as:

$${H}^{\prime}=-{\sum\limits_{i=1}^{L} }{(p}_{i}{\rm{log}}_{2}{p}_{i}),$$

where *L* is the number of response levels (3 or 5 in EQ-5D) and *p*_*i*_ = *n*_*i*_/*N* is the proportion of observations in the *i*th level. ‘*H*’ represents the absolute amount of informativity. The value of ‘*H*’ ranges from 0 (the weakest discriminatory power) to ‘*H*’_max_ for a uniform distribution equal to log_2_*L*. ‘*H*’_max_ is different for EQ-5D-3L (1.58; *L* = 3) and EQ-5D-5L (2.32; *L* = 5). The 95% confidence intervals for ‘*H*’ were calculated based on the variance of the Shannon index [51]:

$${{\rm{var}}\, H}^{\prime}=\frac{{\sum\nolimits_{i=1}^{L} }{{p}_{i}({\rm{log}}_{2}{p}_{i})}^{2}-{\left({\sum\nolimits_{i=1}^{L} }{p}_{i}{\rm{log}}_{2}{p}_{i}\right)}^{2}}{N}$$

Since the range of values for ‘*H*’ depends on the levels of possible answers, the parameter which can objectively determine the relative informativity power, the Shannon Evenness index (‘*J*’), is defined as:

$${J}^{\prime}=\frac{{H}^{\prime}}{{H}^{\prime}{\rm{max}}},$$

where ‘*J*’ = 0 signifies the weakest discriminatory power, when all results are clustered around one answer, and ‘*J*’ = 1 signifies the highest discriminatory power when results are evenly distributed among all the levels of an answer.

Construct validity was analysed in two ways. In the first instance, we analysed it using the method of known-groups validation. For this purpose, we have implemented the analysis of variance model (ANOVA) to explore statistically significant relationships between the EQ-5D index values and age groups (18–29, 30–39, 40–49, 50–59, 60–69, 70+), sex and declared diagnosis of diabetes. We hypothesised that lower values of HRQoL would be more common among older respondents, females and patients having declared diabetes, as reported in studies [52–54]. We used linear regression models, for both sexes, to determine the relationship between age and the EQ-5D index values. We also calculated the Spearman coefficients to estimate the correlation between the domains of EQ-5D-3L and EQ-5D-5L, and with the results of EQ VAS and the first question from the SF-12 questionnaire (SF-1: “Is your health: excellent, very good, good, fair, or poor?”) [55].

**Fig. 1:**
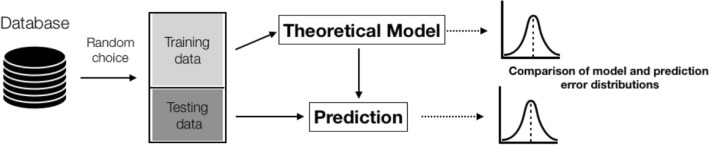
The scheme of the novel approach for construct validity assessment as based on the theoretical model

We developed the construct validity assessment by building a theoretical model of the hypothesised relationship between the characteristics of the respondent and the EQ-5D-5L index values, and then comparing the results of the EQ-5D-5L questionnaire with the prediction of the theoretical model. We designed an input–output model that receives a set of five input parameters (age group, sex, level of education, economic status and occurrence of diabetes) and can assign them the expected EQ-5D-5L index value. This model is based on a machine learning algorithm known as *random forest* [56, 57], commonly used in recent years in biomedical engineering [58–60]. The algorithm works by randomly selecting a subset of input data from the training set and learning a single regression tree, which forms the so-called *random forest*. This scheme is repeated as many times as there are trees in the random forest. The final prediction of the model is the average of the results from an individual tree. To teach the model, we used 60% of the data obtained from the interview database. The model consists of 300 trees, which is a compromise between the expected accuracy of prediction and the complexity of the algorithm. The remaining 40% of the interviews (test data set) is used to determine the convergence of the EQ-5D-5L index values from the survey with the results from the previously built model. For this purpose, we calculated the root-mean-square error measure (RMSE_val_). We also calculated RMSE for the training data set, based on which the model was built, thus determining the internal error of the theoretical model itself (RMSE_int_). Then, we compared the internal error distribution for the model itself, along with the RMSE results for the test data (Fig. 1). In our opinion, the convergence of both errors can infer the construct validity of the EQ-5D-5L questionnaire.

### Statistical analysis

We estimated descriptive statistics to characterise the study population. Pearson’s chi-square test statistic was used to determine statistical significance at ceiling effect levels between EQ-5D-3L and EQ-5D-5L. One-way ANOVA was implemented for all of the parameters under consideration. The linear regression model used the least-squares method. Microsoft Excel (Microsoft® Office 365) and R software (3.6.2) [61] (with external packages [62–65]) were used for the analysis.

## Results

The study was conducted on a representative sample of the adult general population of Poland (*N* = 3978; aged 18–87, 53.2% female; Table 1) [66, 67]. The socio-economic and demographic characteristics of the study population seems to be consistent with the characteristic of the Polish adult population from 2014. Diagnosis of diabetes was declared by 6.8% of respondents. Missing values were found in 0.4%, 0.9% and 0.1% of EQ-5D-5L, EQ-5D-3L and EQ VAS questionnaires, respectively.

**Table 1:** Socio-economic and demographic characteristics of study population, compared with Polish adult population

Characteristic	Study sample (*N* = 3978)		Polish adult population [47, 48, 66, 67] (*N* = 31,500,297)*
	*N*	%	%
Age group (years)			
18–29	765	19.2	20.8
30–39	642	16.1	19.8
40–49	611	15.4	15.5
50–59	701	17.6	17.6
60–69	758	19.1	14.0
70+	501	12.6	12.3
Sex			
Female	2117	53.2	52.3
Male	1861	46.8	47.7
Place of residence			
Rural area	1419	35.7	39.6
Town of < 100,000 inhabitants	1470	37.0	32.2
City of 100,000 and more inhabitants	1089	27.4	28.2
Geographical location of residence (macro-region)			
Central	768	19.3	20.4
Southwest	446	11.2	10.3
South	877	22.0	20.8
Northwest	625	15.7	16.0
North	573	14.4	15.0
East	689	17.3	17.5
Education			
Primary or middle school	680	17.1	23.2
Vocational school	989	24.9	21.7
Secondary school	1310	32.9	29.0
Post-secondary school	119	3.0	2.6
Higher	848	21.3	17.0
Others	32	0.8	6.5
Employment status			
Employed/self-employed	1976	49.7	46.3
Unemployed (able to work)	333	8.4	No data
Unemployed (unable to work, annuitant)	265	6.7	5.6
Student (full time)	285	7.2	6.4
Homemaker, housewife	137	3.4	No data
Retired person	982	24.7	22.0
Number of persons in a household, mean, SD	3.1	1.5	2.8
Considering himself/herself as a religious person	3675	92.4	88.9
General perception of health (SF-1)			
Excellent	245	6.2	7.1
Very good	1007	25.3	30.7
Good	1759	44.2	45.2
Fair	810	20.4	15.2
Poor	155	3.9	1.8
EQ VAS, mean (SD)	74.9	38.9	79.9 (16.9)
Household income (monthly, per person, €)			
≤ 120	397	10.0	Average 340
121–180	382	9.6	
181–240	549	13.8	
241–360	709	17.8	
> 360	859	21.6	
Hard to say	348	8.7	
No response	734	18.5	
Diabetes	272	6.8	8.0 [85]

*2014 census data

The level 1 responses from EQ-5D-3L (3L_1_) were mostly (92.3%; here and later—on average) replaced by level 1 from EQ-5D-5L (5L_1_, Table 2). The 3L_2_ were distributed into 5L_2_ (49.2%), 5L_3_ (35.7%) and 5L_4_ (15.1%). The most uniform distribution of transitions from 3L_2_ to 5L_2_, 5L_3_ and 5L_4_ was observed for the mobility dimension. The 3L_3_ responses were distributed into 5L_4_ (64.5%) and 5L_5_ (35.5%). The highest proportion of transitions from 3L_3_ to 5L_4_ was observed for the pain/discomfort dimension (87.5%). Only for the mobility domain did the proportion of redistribution 3L_3_ to 5L_5_ prevailed (62.5%). According to the definition of Janssen [25], the average percentage of inconsistent transitions of results from EQ-5D-3L to EQ-5D-5L was 4.4% (Table 2).

**Table 2:** Redistribution of the results from the EQ-5D-3L questionnaire to EQ-5D-5L and average inconsistency

Dimension	EQ-5D-3L	EQ-5D-5L	*n*	%	Inconsistency (%)
Mobility	1	1	2857	95.5	3.3
		2	136	4.5	
	2	2	304	37.7	
		3	270	33.5	
		4	233	28.9	
	3	4	9	37.5	
		5	15	62.5	
Self-care	1	1	3521	99.0	2.5
		2	37	1.0	
	2	2	127	46.7	
		3	109	40.1	
		4	36	13.2	
	3	4	12	60.0	
		5	8	40.0	
Usual activities	1	1	3066	96.9	5.8
		2	99	3.1	
	2	2	262	52.1	
		3	178	35.4	
		4	63	12.5	
	3	4	29	67.4	
		5	14	32.6	
Pain/discomfort	1	1	1780	85.6	4.8
		2	300	14.4	
	2	2	781	48.8	
		3	617	38.5	
		4	204	12.7	
	3	4	70	87.5	
		5	10	12.5	
Anxiety/depression	1	1	2172	84.7	5.7
		2	391	15.3	
	2	2	685	60.8	
		3	349	31.0	
		4	93	8.3	
	3	4	26	70.3	
		5	11	29.7	

The best health state (“11111”) was reported by 38.0% and 46.6% of the respondents, when answering the EQ-5D-5L and EQ-5D-3L questionnaires, respectively (*p* < 0.01, Table 3). The highest ceiling effect was observed for the self-care dimension, both for EQ-5D-3L and EQ-5D-5L. The estimated ceiling effects for individual dimensions of EQ-5D-5L and EQ-5D-3L were not statistically different.

**Table 3:** Comparison of the ceiling effect results in the EQ-5D-3L and EQ-5D-5L dimensions

Dimension	Ceiling effect		Change from EQ-5D-3L to EQ-5D-5L (%)	*p* value
	EQ-5D-3L (%)	EQ-5D-5L (%)		
Mobility	74.3	73.7	0.6	NS
Self-care	90.9	90.3	0.5	NS
Usual activities	82.6	82.1	0.5	NS
Pain/discomfort	47.8	47.2	0.6	NS
Anxiety/depression	58.5	57.9	0.6	NS
Health state (“11111”)	46.6	38.0	8.7	*p* < 0.01

*NS* not significant

Shannon’s index was largest for the pain/discomfort dimension, and smallest for the dimension of self-care, both for the EQ-5D-3L and EQ-5D-5L questionnaires (Table 4). For the EQ-5D-5L questionnaire, an increase in relative informativity power was observed for the dimensions of anxiety/depression, pain/discomfort and mobility and to a greater degree, a decrease in relative informativity power (17.9% and 19.3%) was observed for the dimensions of usual activities and self-care, respectively.

**Table 4:** Informativity power results for the EQ-5D-3L and EQ-5D-5L questionnaires

Dimension	*H′*			95% CI *H*′			*J*′		% change *J*′ from EQ-5D-3L to EQ-5D-5L
	EQ-5D-3L	EQ-5D-5L		EQ-5D-3L	EQ-5D-5L		EQ-5D-3L	EQ-5D-5L	
Mobility	0.825	1.253		0.825–0.826	1.253–1.254		0.522	0.540	3.5
Self-care	0.487	0.587		0.487–0.488	0.587–0.588		0.308	0.253	− 17.9
Usual activities	0.787	0.932		0.786–0.787	0.931–0.933		0.498	0.402	− 19.3
Pain/discomfort	1.139	1.765		1.139–1.140	1.764–1.765		0.721	0.761	5.5
Anxiety/depression	1.004	1.495		1.004–1.004	1.495–1.496		0.635	0.645	1.4

*CI* confidence interval, *H′* Shannon index, *J′* Shannon Evenness index

The results of the known-groups validity for the EQ-5D-5L and EQ-5D-3L questionnaires are summarised in Table 5. The analysis of variance indicates statistically significant differences in the results of the EQ-5D index values for six age groups, sex (lower utility score in women) and declared diagnosis of diabetes (lower utility score in diagnosed diabetes), both for EQ-5D-5L and EQ-5D-3L, which confirms the previous hypothesis. A reduction of EQ-5D-5L index value with age was observed, and this tendency was noted by the negative linear trend in the regression models:

$${\rm{for\, males}}{:}\; {\rm{EQ}}{\text{-}}5{\rm{D}}{\text{-}}5{\rm{L}}_{\rm{index\, value}} = -0.0029*{\rm{age}} + 1.0674$$

$${\rm{for\, females}}{:}\; {\rm{EQ}}{\text{-}}5{\rm{D}}{\text{-}}5{\rm{L}}_{\rm{index\, value}} = -0.0036*{\rm{age}} + 1.0903$$

**Table 5:** Known-groups validity results of the EQ-5D-5L and EQ-5D-3L questionnaires

Known-group	Mean EQ-5D index		ANOVA *F*-statistic		*p* value	
	5L	3L	5L	3L	5L	3L
Age group (years)						
18–29	0.981	0.967	166.37	241.04	*p* < 0.001	*p* < 0.001
30–39	0.971	0.952				
40–49	0.938	0.931				
50–59	0.899	0.881				
60–69	0.869	0.836				
70+	0.832	0.761				
Sex						
Female	0.903	0.883	19.95	33.56	*p* < 0.001	*p* < 0.001
Male	0.935	0.905				
Diabetes						
Yes	0.908	0.751	84.15	118.41	*p* < 0.001	*p* < 0.001
No	0.923	0.903				
SF-1						
Excellent	0.997	0.991	532.70	463.60	*p* < 0.001	*p* < 0.001
Very good	0.987	0.977				
Good	0.955	0.920				
Fair	0.818	0.776				
Poor	0.544	0.491				

In Table 6, we collected the results of the convergent validity assessment between the EQ-5D-5L questionnaire and EQ VAS, EQ-5D-3L and SF-1. In most cases, we observed a moderate correlation. The results for the same dimensions from EQ-5D-5L and EQ-5D-3L were convergent (*italic* in Table 6). Mobility and pain/discomfort are the dimensions most correlated with EQ VAS and SF-1.

**Table 6:** The convergent validity results for correlation of EQ-5D-5L with EQ VAS, EQ-5D-3L and SF-1 (Spearman coefficients results, all statistically significant)

Dimension		EQ VAS	EQ-5D-3L					SF-1
			Mobility	Self-care	Usual activities	Pain/discomfort	Anxiety/depression	
EQ-5D-5L	Mobility	− 0.53	*0.83*	0.52	0.63	0.53	0.37	0.56
	Self-care	− 0.36	0.48	*0.78*	0.56	0.33	0.33	0.39
	Usual activities	− 0.47	0.60	0.62	*0.73*	0.45	0.38	0.49
	Pain/discomfort	− 0.60	0.56	0.40	0.51	*0.78*	0.48	0.64
	Anxiety/depression	− 0.48	0.39	0.33	0.39	0.48	*0.70*	0.49
	EQ-5D-5L index	0.66	− 0.65	− 0.45	− 0.58	− 0.76	− 0.58	− 0.68

*Italic* means results of the same dimensions

### The random forest algorithm for construct validity

**Fig. 2:**
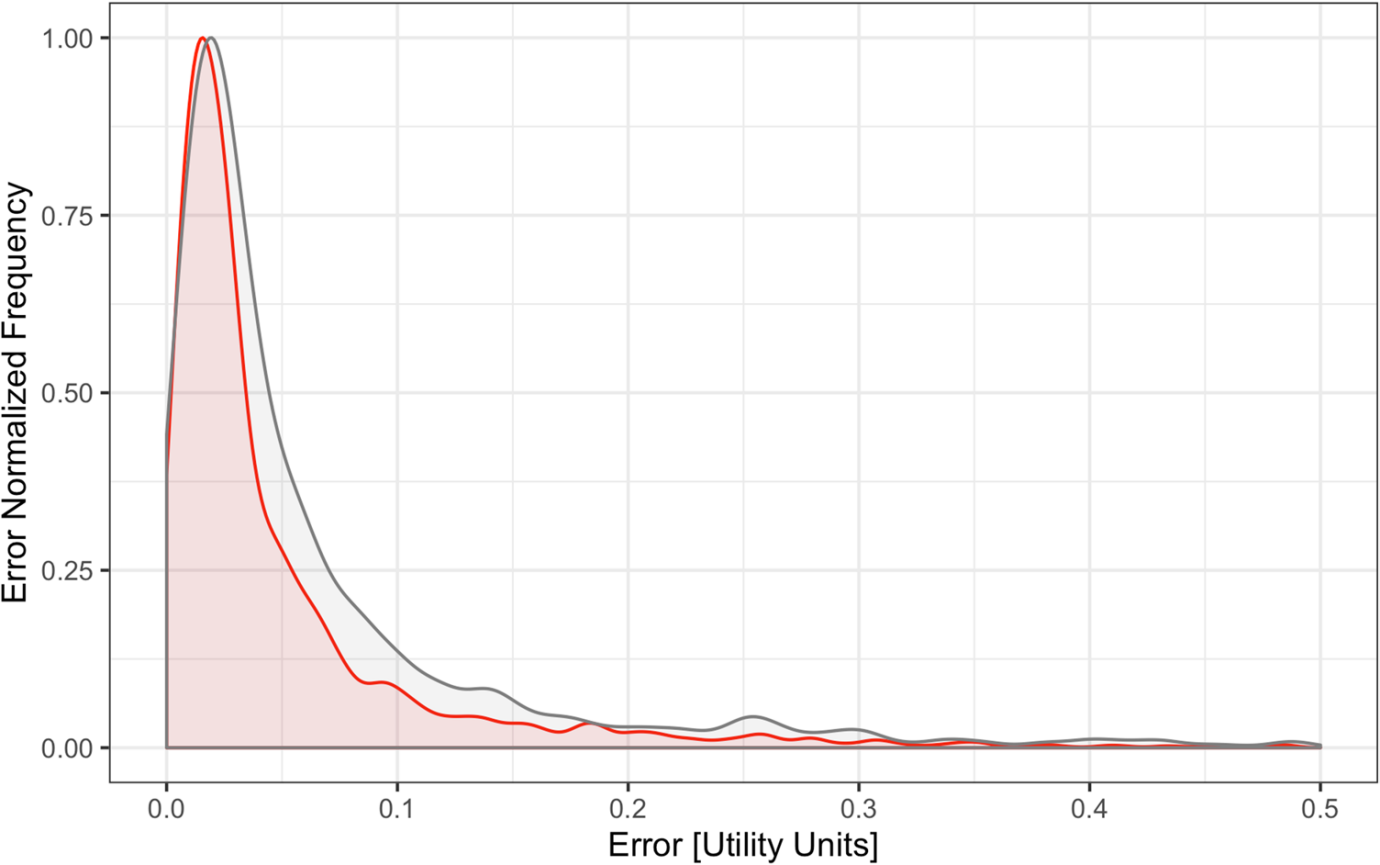
The comparison of the model internal error (red) and testing data set prediction error (grey) distributions. (Color figure online)

The theoretical model which can predict what the HRQoL of a patient with a specific characteristic will look like, was constructed on the basis of the random forest algorithm, which is widely used in machine learning. For the assessment of construct validity, we wanted to compare the results of prediction HRQoL from the model with the results from the survey. The internal error of the theoretical model (RMSE_int_ for the training data set) was 0.095 in utility units. This means that the model, using the same data set from which it was taught, makes an average prediction error of < 0.1 utility units. The RMSE_val_ measure was equal to 0.121. This was calculated for the difference between the prediction of the theoretical model and the testing data set. In Fig. 2, we present the comparison of the theoretical model error distribution with the error distribution for the test data set. It can be seen that these distributions overlap almost completely. The estimations in Table 7 indicate that 85.6% of the errors for the test data set were lower than the 95th quantile of the theoretical model error distribution. On this basis, we can state that the results obtained from the EQ-5D-5L questionnaire (EQ-5D-5L index value) are consistent with the theory (developed from the model), which suggests a positive assessment of the construct validity of the EQ-5D-5L questionnaire.

**Table 7:** The construct validity assessment results, based on the theoretical model

	RMSE	95th quantile from the distribution of model error results	% validation results below 95th quantile of model errors
Theoretical model (random forest)	0.095	0.125	85.6%
Validation	0.121		

*RMSE* root-mean-square error

## Discussion

According to our best knowledge, this is the first study reporting the validity of the EQ-5D-5L in comparison to EQ-5D-3L, measured on the same sample of the general population coming from a European country. A novel approach for construct validity assessment was proposed, based on the machine learning algorithm known as the *random forest*.

We identified other studies from Greece [72] and Spain [51], in which authors compared the EQ-5D-5L and EQ-5D-3L in the same sample, but they include limitations. The Greek study population was from Athens and in age over 40, so this not constitute the general population of a country. In the Spanish study, the sample was from Catalonia. Both analyses used the mapping methods for calculation of the country value sets. In our study, for an estimate of both the EQ-5D-5L and EQ-5D-3L index values, we used directly measured country-specific value sets.

The results of the analysis show that the EQ-5D-5L questionnaire has a lower ceiling effect compared to the EQ-5D-3L version. Redistribution from EQ-5D-3L to EQ-5D-5L was similar for each dimension (i.e. transfer responses from 3L_1_ to 5L_1_ are more common than from 3L_1_ to 5L_2_ [about 90% vs 10%], transfer responses from 3L_2_ to 5L_2_ and 3L_2_ to 5L_3_ are more common than from 3L_2_ to 5L_4_, and transfer responses from 3L_3_ to 5L_4_ are more common than from 3L_3_ to 5L_5_ [about 70% vs 30%]). The mean inconsistency did not exceed 5%. In terms of Shannon's relative informativity power, the discrepancy of the results between each domain does not allow to draw clear conclusions about the advantage of one version of the questionnaire. The results of known-groups validation confirmed the hypothesis about the relationship between the EQ-5D index value and age group, sex and occurrence of diabetes. Individual dimensions of EQ-5D-5L correlate with the assessment of health on the EQ VAS scale, with the domains from EQ-5D-3L and SF-1. The results imply that EQ-5D-5L is a valid instrument for use in the Polish adult population.

The strength of this study lies in the fact that both the EQ-5D-5L and EQ-5D-3L questionnaire and EQ VAS results came from the same study iteration, and all are evaluated on the same occasion in the same group. Often, in studies comparing the properties of EQ-5D-3L and EQ-5D-5L in the general population, the samples for both versions of questionnaires were separate [68, 69]. The recently published study by Thompson et al. is also a kind of “indirect study”. Still, authors used the matching method to join individuals completing the EQ-5D-3L with those completing the EQ-5D-5L, and thus increasing the sample size [70].

We conducted our study on a representative sample of the general adult population of Poland. Therefore, validation of the EQ-5D-5L questionnaire takes into account a wide spectrum of a population characteristic. In the literature, a comparison of EQ-5D-3L and EQ-5D-5L questionnaire properties were often carried out in smaller populations that did not apply to the whole country [51, 69, 71–73]. The exceptions were studies performed in England, Germany, Spain, USA, South Korea, Japan and Malaysia [68, 74–79]. The psychometric properties may also be assessed in studies of value sets for individual countries, although they are often limited to the distribution of responses or the ceiling/floor effects [80, 81]. In our study, we used the directly measured country-specific EQ-5D index value sets for Poland, developed for both versions of the EQ-5D questionnaire (time trade-off (TTO)-based for EQ-5D-3L and TTO/discrete choice experiment (DCE)-based for EQ-5D-5L [47, 48]). This increased the reliability of the presented HRQoL assessment. In other studies, the interim scoring value sets for EQ-5D-5L were used [51, 72, 75, 77] or the EQ-5D index values were not estimated [69, 73, 74, 76, 79]. The directly measured country-specific value sets were used only in one study from Japan [78].

As a part of the construct validity assessment, we proposed a novel approach based on the theoretical model for HRQoL assessment in the Polish population, designed using the *random forest* algorithm for regression. Developing the model based on data obtained from a representative sample of the general population represents an attempt to create a reference for the HRQoL results that depends on the parameters describing the population characteristics. Relating the results of the questionnaire to the HRQoL predicted by the model, and comparing the accuracy of such an assessment with the accuracy of the model itself, allows us to conclude that the questionnaire is convergent with the predicted theory.

One of the limitations of this study may be the inability to assess reliability and responsiveness (similar results in the same study sample for a repeated protocol). Another potential limitation is the lack of construct validity assessment (through known-groups validation) related to multimorbidity. Further, the diagnoses of diabetes in our study are not the result of clinical assessment but were based on self-reporting. The fixed order of presented questionnaires in the survey could pose some limitations (i.e. introduce ordering effect). However, the introduction of the SF-12 questionnaire between the EQ-5D-5L and the EQ-5D-3L questionnaires may somehow eliminate the potential response memory effect.

Similarly to our study, a significant reduction in the ceiling effect for EQ-5D-5L in comparison to EQ-5D-3L was observed in other general population studies [28, 51, 68, 69, 71–73, 76, 77, 79] (Table 8). It is also worth noting that the ceiling effect at the level of 38% for EQ-5D-5L is much lower than the results obtained in other countries, which may be influenced by the characteristics of the studied populations themselves [51, 79]. The higher percentage of missing answers for EQ-5D-3L could be explained by the sequence of questionnaires in the survey (EQ-5D-3L was always the last one to be completed). As expected, an increase in Shannon informativity power was observed for the EQ-5D-5L questionnaire [28, 51, 72, 79]. However, the relative informativity power was often similar or lower than in the EQ-5D-3L questionnaire, which prevents us from claiming that EQ-5D-5L has greater informativity power than EQ-5D-3L, as has been reported in other studies [51, 68, 79]. The results of the study indicated a relatively large percentage of inconsistencies (4.4%), while in other studies it rarely exceeded 2% on average [71, 77]. In Janssen et al. [25], where the definition of inconsistency came from, it was 1.1% with 5912 observations. The results from our survey seem to be quite different from this (4.4% in 3978 observations), which may mean that the respondents to the EQ-5D-3L and EQ-5D-5L questionnaires were not entirely consistent. Perhaps it was related to the fact that the EQ-5D-3L questionnaire was not completed first and not immediately after EQ-5D-5L (EQ VAS and SF-12 were completed in between). Our results for the known-groups validation are convergent with other analyses [71, 72, 77], in particular, with respect to age and gender. No data were found analysing the convergence of the EQ-5D index values with declared diabetes in the general population.

**Table 8:** The ceiling effect results from other validation studies of EQ-5D-5L in the general population

References	Country	Ceiling effect		Change from EQ-5D-3L to EQ-5D-5L (%)
		EQ-5D-3L (%)	EQ-5D-5L (%)	
Yfantopoulos [72]	Greece	47.0	31.2	− 15.8
Shafie [79]	Malaysia	66.4	50.6	− 15.8
Ferreira [71]	Portugal	61.1	46.5	− 14.6
Craig [76]	USA	43.6	32.5	− 11.1
Thompson [70]	UK	54.4	43.8	− 10.6
Agborsangaya [69]	Canada	42.1	32.3	− 9.8
Feng [68]	England	56.2	47.6	− 8.6
Scalone [73]	Italy	43.9	38.0	− 5.9
Kim [77]	South Korea	65.7	61.2	− 4.5
Martí-Pastor [51]	Spain	61.8	60.8	− 1.0

In Henry et al. [82] authors estimated the minimally important difference (MID) for EQ-5D-5L scoring algorithms, including that from the Polish population. The mean result was 0.080 (0.030). The range of MID averages for different countries was from 0.072 (Malaysia) to 0.101 (Taiwan). In Coretti et al. [83] the minimally (clinically) important difference (MCID) for EQ-5D index value ranged from 0.03 to 0.54, with the raw average across 18 studies of 0.18. However, Coretti et al. review was about disease-specific populations. In our construct validity assessment based on the theoretical model the RMSE_val_ (0.121) measure appears to be close to one SD difference above from the average value for the Polish algorithm and within the range described by Coretti et al. Due to the large scatter of the estimated MID it is still difficult to say if RMSE_val_ is large or small. However, in our approach to the construct validity assessment, the most critical aspect is the convergence between the RMSE_val_ and RMSE_int_ rather than the utility results, and in this case, we observed that the errors distributions overlap almost completely (Fig. 2).

In Poland, we found only one validation study of a generic questionnaire (SF-36v2) for the general population of a country [84], and one assessment of the reliability of the generic questionnaire (WHOQOL-BREF) within a local community (Silesian agglomeration [85]). The fact that the EQ-5D validation study has not been available constituted essential unmet research need, as EQ-5D is commonly used among Polish patients and is recommended by the guidelines (Polish Agency for Health Technology Assessment [86]). Our study would support the credibility of the questionnaire and the correct interpretation of its results. Moreover, this study may also support its validity in countries with similar socio-economic characteristics in Central and Eastern Europe.

In the future, it would be worth assessing the reliability and responsiveness of the EQ-5D-5L questionnaire and extending the validity assessment with other parameters of the population characteristics, including multimorbidity.

## Conclusions

In this study, the validity of the EQ-5D-5L questionnaire was assessed in a sample of the general adult population of Poland and, in comparison with EQ-5D-3L, showed similar (informativity power, known-groups validation) or better (ceiling effect) psychometric properties for this population. A novel approach to the assessment of construct validity was proposed, based on the use of the machine learning technique known as the *random forest* algorithm. The comparison of the model predictions and the results of the interviews provided a basis for the positive assessment of the construct validity. EQ-5D-5L is a commonly used instrument in HRQoL assessment, and this study will support the correct interpretation of its results in studies performed among the Polish population.

## Data Availability

Data are available from the author on a reasonable request.
